# Preoperative Predictors of Poor Outcomes Following Lumbar Discectomy. A Study Based on the National Finspine Registry

**DOI:** 10.1097/BRS.0000000000005425

**Published:** 2025-06-13

**Authors:** Antti Saarinen, Eetu Suominen, Liisa Pekkanen, Antti Malmivaara, Jukka Huttunen, Katri Pernaa, Henri Salo, Jussi P. Repo

**Affiliations:** a Department of Orthopedics and Traumatology, Turku University Hospital and University of Turku; b Research Unit, Orton Orthopedic Hospital, Helsinki; c Department of Orthopaedics and Traumatology, Hospital Nova of Central Finland, Jyväskylä; d Department of Knowledge Brokers, Finnish Institute for Health and Welfare, Helsinki; e Department of Neurosurgery, Kuopio University Hospital and Institute of Clinical Medicine, University of Eastern Finland, Kuopio; f Department of Orthopaedics and Traumatology, Tampere University Hospital, Tampere, Finland

**Keywords:** discectomy, poor outcome, register

## Abstract

**Study Design.:**

Retrospective cohort study.

**Objective.:**

To identify predictors for poor outcome after lumbar discectomy for herniated disc.

**Background.:**

Lumbar discectomy for herniated disc is a common spinal procedure. Despite the surgical treatment, some patients are left with persistent pain and poor health-related quality of life. We aim to research preoperative predictive factors associated with poor outcome after lumbar discectomy.

**Materials and Methods.:**

National Spine Surgery Registry was searched for patients who underwent primary discectomy for lumbar disc herniation between 2017 and 2022. All patients had a minimum of 2 years of follow-up. The primary outcome was disability at 12 months postoperatively, assessed using the Oswestry Disability Index (ODI). Patients were categorized into satisfactory (ODI: 0–40) and poor outcome groups (ODI: 41–100). Logistic regression was used to identify preoperative predictors of poor outcome. Variables for multivariable analysis were selected based on clinical relevance assessed by senior authors and bivariate associations. Secondary outcomes included pain scores and patient-reported satisfaction.

**Results.:**

In all, 3339 patients were included, of whom 2991 (90%) had minimal to moderate disability and 348 (10%) had severe disability assessed with ODI at the follow-up. Several factors were identified to associate with poor outcome after the surgery: older age (OR: 1.03, 95% CI: 1.02–1.03), female sex (OR: 1.28, 95% CI: 1.03–1.61), higher body mass index (OR: 1.06, 95% CI: 1.02–1.09), cardiologic comorbidity (OR: 4.27, 95% CI: 2.4–7.3), regular preoperative painkiller use (OR: 2.2, 95% CI: 1.5–3.3), and higher number of operated vertebrae (OR: 2.4, 95% CI: 1.6–3.6). Symptom lasting over 1 year was associated with worse outcomes when compared with symptoms for 3 to 12 months (OR: 0.42, 95% CI: 0.29–0.60), 6 to 12 weeks (OR: 0.23, 95% CI: 0.12–0.39), and those with symptoms for <6 weeks (OR: 0.35, 95% CI: 0.19–0.62). Employed individuals were significantly associated with better outcomes when compared other statuses. Worse preoperative quality of life scores were associated with poor outcome.

**Conclusion.:**

Several preoperative factors were associated with poor outcome after lumbar discectomy. Identifying higher-risk patients—such as those with high BMI, older age, or significant comorbidities—can support preoperative counseling and targeted interventions. Optimizing modifiable factors preoperatively may improve outcomes.

Lumbar disc herniation is common in the adult population and primarily resulting from degenerative changes. Lumbar herniation can cause nerve root compression and lower limb radiculopathy. Most of the patients with lumbar herniation recover with conservative method consisting of sufficient painkillers and medical therapy.^1^ In cases of extreme radiculopathy, paresis, or cauda equina syndrome surgical treatment is often considered. Early surgical treatment for lumbar disc herniation may lead to faster recovery with improved cost-effectiveness when compared with conservative treatment with similar clinical results at 1 year of follow-up.^2^

Lumbar discectomy for herniated disc is one of the most common spinal procedures.^3^ Complications of lumbar discectomy are relatively rare, including dural lesion, infection, and hematoma.^4^ While outcomes of lumbar discectomy are generally satisfactory, up to a third of the patients are left with persistent pain after the surgical treatment.^5–7^ Various clinical and socioeconomic predictors for poor outcomes after lumbar discectomy have been reported, such as intact annulus fibrosus, greater severity of symptoms, and longer duration of sick leave.^8^ A systematic review of 40 studies identified preoperative leg pain, positive mental health status, shorter duration of symptoms, and younger age to associate with better outcomes.^8^ It is essential to clarify which factors could lead to worse outcomes to communicate the potential benefit for surgery to the patients.

The Finnish spine register (FinSpine) is a national register collecting prospectively data from all the Wellbeing Services Counties.^9^ We report factors associated with satisfactory and poor outcome after discectomy for lumbar disc herniation from this national spine surgery database.

## MATERIALS AND METHODS

A prospectively collected national spine surgery register was searched for patients who underwent primary discectomy for lumbar disc herniation between the years 2017 and 2022. In our country, health care is publicly funded and affordable, ensuring that spine surgery is accessible to all patients. Preoperative patient-reported characteristics and outcome measures were collected. Data included in the FinSpine register including specifications of perioperative and postoperative complications are presented in a paper by Marjamaa *et al* ^9^ in more detail.

Outcome was assessed using the Oswestry Disability Index (ODI), which is a validated patient-reported outcome instrument developed for assessing the impact of back pain on the activity and daily living.^10^ ODI scores ranging between 0 and 40 points indicate minimal to moderate disability, and scores from 41 to 100 are interpreted from severe disability to bedbound. The primary outcome was the ODI score assessed at 12 months postsurgery. ODI score can be classified into 5 categories: 0 to 20 as minimal, 21 to 40 as moderate, 41 to 60 as severe disability, 61 to 80 as crippled, and 81 to 100 as bedbound or exaggerating.^11^ Minimal to moderate disability (0–40) was classified as satisfactory outcome and severe disability (41–100) as poor outcome. Secondary outcomes were general pain, pain in shoulders, lumbar spine, upper extremity, and lower extremity assessed with Visual Analog Scale (VAS), and patient-reported satisfaction. Patients were asked if they were satisfied with the postoperative status (“I am satisfied,” “I am not satisfied,” “I am not sure”), would they undergo the surgery again (“Yes,” “I am not sure,” “No”), and how they perceived the postoperative status (“Worse than before the surgery,” “Better,” “No change”). Outcome variables were gathered 0 to 2 months before the surgery, 3 (2–4) months after the surgery, 12 (11–13) months after the surgery, and at 24 (23–25) months after the surgery.

Distribution of the data was analyzed using Q-Q plots and the Shapiro-Wilk test. Skewed data are presented with medians and interquartile ranges (IQRs). Correlation analyses for multicollinearity were performed, and no strong correlations were found (correlation >0.7). All senior authors were asked to evaluate the clinical relevance of the preoperative characteristics used in the bivariate analysis on a 1 to 5 scale. Characteristics were included in the multivariable model if at least 50% of the senior authors considered them to be somewhat important or very important predictors. The multivariable model included the following covariates: age, pain score at 12 months, preoperative cauda equina syndrome, pain medication use, and cardiologic comorbidity. Odds ratios (ORs) with 95% CIs were calculated to determine the strength and significance of associations. A *P*-value <0.05 was considered statistically significant. The performance of the model was assessed using the area under the receiver operating characteristic curve (AUC).

## RESULTS

**Figure 1:**
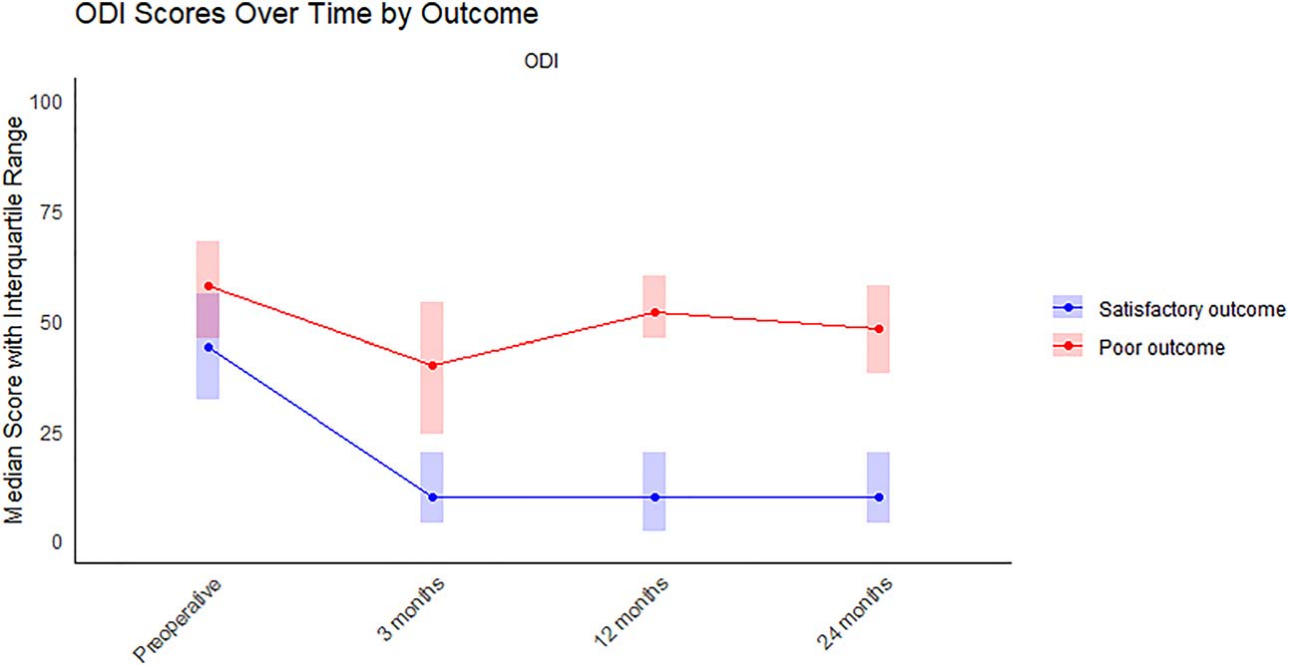
Oswestry Disability Index scores during the follow-up. Higher score refers to worse outcome.

In all, 8231 patients treated in 19 hospitals and having undergone discectomy for lumbar herniation were identified from the database. A minimum of 12 months follow-up with ODI was available from 3339 patients who underwent primary surgery. Of these patients, 2991 (90%) had minimal to moderate disability (satisfactory group) and 348 (10%) had severe disability (poor outcome group), as assessed using ODI (Table 1). The median (IQR) age was 44 (22) years in the satisfactory group and 51 (23) in the poor outcome group. Median (IQR) improvement of ODI score from preoperative to 12 months postoperative was 32 (26) in the satisfactory outcome group and 4 (23) in the poor outcome group (*P* < 0.005, Figure 1). At the last available follow-up, 18.5% reported the best score in the satisfactory group, that is reached the ceiling effect. There was no ceiling or floor effect present in the poor outcome group.

**TABLE 1:** Clinical and Sociodemographic Characteristics With Bivariate Associations to the Primary Outcome (ODI at 12 mo)

	Satisfactory Outcome (n = 2991); n (%)	Poor Outcome (n = 348); n (%)	Odds Ratio	Lower 95% CI	Upper 95% CI
Female	1403 (46.9) [100]	185 (53.2) [100]	1.38	1.03	1.61
Age (median)	44 (22) [100]	50.5 (23) [100]	1.03	1.02	1.03
Body mass index (median)	27.08 (5.865) [59]	28.28 (7.41) [46]	1.06	1.02	1.09
Cauda	102 (3.48) [98]	20 (5.81) [99]	1.71	1.02	2.75
Employment status	59%	47%	—	—	—
Employed	1021 (58.6)	48 (29.8)	Reference	—	—
Unemployed	127 (7.3)	26 (16.2)	4.35	2.58	7.21
Disabled	376 (21.6)	45 (28.0)	2.55	1.66	3.89
Pensioner	217 (12.5)	42 (26.1)	4.12	2.65	6.39
Nicotine use	469 (26.6) [59]	51 (31.5) [46]	1.27	0.89	1.79
Painkiller use	1195 (67.7) [59]	135 (81.8) [47]	2.15	1.45	3.29
Cardiologic comorbidity	55 (3.7) [50]	19 (14.1) [39]	4.27	2.40	7.32
Pain duration	59%	47%	—	—	—
Pain <6 wk	218 (12.4)	15 (9.1)	0.35	0.19	0.62
Pain 6–12 wk	343 (19.4)	15 (9.1)	0.23	0.12	0.39
Pain 3–12 mo	869 (49.2)	70 (42.4)	0.42	0.29	0.60
Pain over year	335 (19.0)	65 (39.4)	Reference	—	—
Perioperative complications	105 (3.6)	17 (5.0)	1.42	0.81	2.34
Postoperative complications	51 (3.5)	13 (8.1)	2.44	1.25	4.46

Reference: variable used as the reference value in the analysis.

Proportion of the sample with available data in brackets [].

Several preoperative characteristics were significantly associated with poor outcome (ODI score 41 or higher) after surgical treatment (Table 1). Older age (OR: 1.03, 95% CI: 1.02–1.03), female sex (OR: 1.28, 95% CI: 1.03–1.61), higher body mass index (OR: 1.06, 95% CI: 1.02–1.09), preoperative cauda equina syndrome (OR: 1.7, 95% CI: 1.0–2.7), regular preoperative painkiller use (OR: 2.2, 95% CI: 1.5–3.3), and cardiologic comorbidity (OR: 4.27, 95% CI: 2.4–7.3) were associated with poor outcomes. Patients aged 65 years or older had 2.12 times higher odds of a poor outcome compared with younger patients (95% CI: 1.60–2.78). Patients with BMI ≥25 had 1.44 times higher odds of a poor outcome compared with those with lower BMI (95% CI: 1.00–2.13). Using patients with symptoms lasting over 1 year as the reference group, those with symptoms for 3 to 12 months (OR: 0.42, 95% CI: 0.29–0.60), 6 to 12 weeks (OR: 0.23, 95% CI: 0.12–0.39), and those with symptoms for <6 weeks (OR: 0.35, 95% CI: 0.19–0.62). Using employed individuals as the reference group, those on a pension (OR: 4.1, 95% CI: 2.6–6.4), unemployed individuals (OR: 4.4, 95% CI: 2.6–7.2), and disabled individuals (OR: 2.5, 95% CI: 1.7–3.8) had significantly worse outcomes. Using L5-S1 as the reference level, L4-L5 was not significantly associated with worse outcomes (OR: 1.3, 95% CI: 0.98–1.8), while L3-L4 (OR: 1.9, 95% CI: 1.2–2.9) and L2-L3 (OR: 2.4, 95% CI: 1.3–4.2) were associated with poor outcomes. In addition, a higher number of operated disc spaces was linked to worse outcomes (OR: 2.4, 95% CI: 1.6–3.6). Perioperative complications were not significantly associated on outcome (OR: 1.4, 95% CI: 0.81–2.3), while postoperative complications were associated with poor outcome (OR: 2.4, 95% CI: 1.2–4.5).

**Figure 2:**
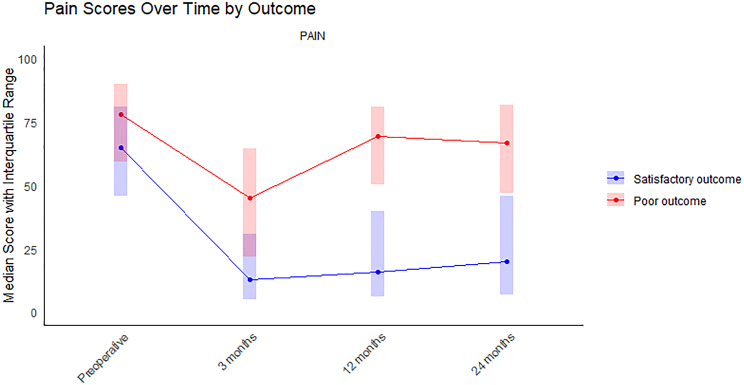
Patient reported general pain scores during the follow-up. Pain measured using Visual Analog Scale (VAS). Higher score refers to worse outcome.

Worse preoperative ODI, general pain VAS (Figure 2), lower back pain, and lower extremity pain scores were associated with poor outcome (Table 2). Shoulder pain and upper extremity VAS scores were significantly higher in the poor outcome group. ODI scores remained lower in all follow-up time points in the poor outcome group when compared with the satisfactory outcome group.

**TABLE 2:** Patient-Reported Outcomes and Bivariate Associations With the Primary Outcome (ODI at 12 mo)

	Satisfactory Outcome (n = 2991); n (%)	Poor Outcome (n = 348); n (%)	Odds Ratio	Lower 95% CI	Upper 95% CI
ODI score	44 (24) [59]	58 (22) [48]	1.04	1.03	1.05
ODI score at 3 mo	10 (16) [60]	40 (30) [51]	1.08	1.07	1.09
ODI score at 12 mo	10 (18) [100]	52 (14) [100]	NA	—	—
ODI score at 24 mo	10 (16) [57]	48 (20) [60]	1.13	1.12	1.15
ODI change minimum of 15 points	1453 (81.72)	37 (22.29)	0.1	0.04	0.09
Pain	65 (35) [58]	78 (30.5) [46]	1.02	1.01	1.03
Pain 3 mo	13 (26) [57]	45 (42.5) [49]	1.03	1.03	1.04
Pain 12 mo	16 (34) [81]	69.5 (30.75) [82]	1.1	1.05	1.07
Pain 24 mo	20 (39) [55]	67 (35) [59]	1.05	1.04	1.05
Pain decreased minimum of 50%	1083 (64.73)	16 (10.19)	0.1	0.04	0.10

Reference: variable used as the reference value in the analysis.

Proportion of the sample with available data in brackets [].

In the multivariable logistic regression analysis, older age (OR: 1.02, 95% CI: 1.00–1.04), higher pain scores at 12 months (OR: 1.08, 95% CI: 1.06–1.09), presence of cardiologic comorbidity (OR: 4.6, 95% CI: 2.0–10.4), and preoperative pain medication use (OR: 3.2, 95% CI: 1.8–6.3) were independently associated with poor outcomes (Table 3). Preoperative cauda equina syndrome was not significantly associated with poor outcome in the adjusted model (OR: 0.45, 95% CI: 0.02–3.8). The model demonstrated excellent discrimination, with an area under the curve of 0.94.

**TABLE 3:** Results of the Multivariable Logistic Regression Analysis Evaluating the Association Between Preoperative Patient Characteristics and Poor Outcome

Predictor	Odds ratio	Lower 95% CI	Upper 95% CI
Age	1.02	1.01	1.04
Pain at 12 mo	1.07	1.06	1.08
Cauda equina	0.45	0.02	3.77
Preoperative painkiller	3.23	1.77	6.27
Cardiologic comorbidity	4.63	2.04	10.42

Variables included in the multivariable logistic regression model were selected based on clinical relevance as assessed by senior authors. Clinical relevance was determined by expert rating, and variables rated as somewhat or very important by at least 50% of senior authors.

## DISCUSSION

Our study identified several preoperative factors that were associated with poor outcome after discectomy for lumbar disc herniation. In our study, older age, female sex, higher BMI, cardiological comorbidity, duration of the symptoms >12 months, employment status and higher usage of continuous pain medication were all associated with negative outcome after surgery.

In the present study, 10% of patients undergoing primary lumbar discectomy were left with poor outcome. The improvement of the ODI during the follow-up was significantly lower in patients who were left with poor outcomes. In these patients, ODI scores improved from preoperative to 3 months follow-up but remained deteriorated at 12-month and 24-month follow-up. In the satisfactory outcome group, postoperative ODI scores remained low during the whole of the follow-up. This aligns with previous findings suggesting that PROMs show minimal change between 1-year and 2-year follow-up in patients undergoing elective surgery for degenerative spine conditions, indicating that a 1-year follow-up period may be sufficient to assess surgical outcomes.^12^

In a study by Suri *et al* ^6^ concerning secondary analysis of a concurrent randomized trial and cohort study, the risk of recurrent leg pain after lumbar discectomy was 20% and 45% at 1 and 3 years postoperatively. Complete postoperative leg pain resolution, smoking, and depression were identified as predictors for recurrent leg pain in a multivariable analysis.^6^ In our study, the leg pain decreased significantly more in the satisfactory group and remained low during the follow-up. Shoulder pain and upper extremity pain are not associated with lumbar disc herniation. In our study, patients with satisfactory outcome did not report marked shoulder or upper extremity pain when compared with significantly higher scores in the poor outcome group. This may indicate that in some patients the etiology of the pain is other than the herniated lumbar disc, rendering lumbar discectomy ineffective to the general pain as other sites of pain persist.

Symptoms from lumbar disc herniation are common in the adult population. Conservative treatment is recommended on most cases, with cauda equina syndrome, progressive paresis, and intolerable pain being considered as indications for surgery as the primary treatment. Surgical treatment may be considered after symptoms persist despite conservative treatment. In our study, longer symptom duration was associated with worse outcomes. Persistent pain despite conservative treatments may be caused by factors also other than the prolapsed disc, especially in patients with pain not specifically focused on the lower extremity. Previous studies have consistently shown a strong association between symptom duration of 6 months or less and positive postoperative results.^8,13^ We focused on primary lumbar discectomy outcomes without including reoperations in the analysis. Previous research has shown that additional surgery after primary lumbar disc prolapse surgery is relatively common. The most frequent reason for reoperation was recurrent disc prolapse, followed by spinal stenosis and segmental pain. According to the previous study, health-related outcomes tended to deteriorate after multiple additional operations.^14,15^

In our study, higher BMI, older age, and female sex were associated with poor outcomes. The previous evidence has been conflicting according to a meta-analysis.^8^ The previous meta-analysis presented smoking as a conflicting predictor for outcome after lumbar discectomy, with 4 included studies presenting smoking as a negative predictor and 2 as not significant predictor.^8^ In our study, the proportion of patients reporting nicotine use was higher in the poor outcome group (32%) when compared with the satisfactory outcome group (27%), but this difference was not statistically significant. Similar to our findings, the meta-analysis identified better mental health status as a positive predictor and worker’s compensation and longer duration of sick leave as negative predictors.^8^ Prior research has demonstrated that elderly patients tend to have worse postoperative outcomes following lumbar disc herniation surgery, with less improvement in PROM scores and lower satisfaction compared with younger patients.^16^ Patient-reported cardiologic comorbidity had the strongest association with poor outcomes in the present study, which may be related to the overall poorer health status of these patients.

In our multivariable analysis, older age, higher pain scores at 12 months, cardiological comorbidity, and preoperative pain medication use remained significant predictors of poor outcomes. Persistent pain at 12 months had the strongest association, emphasizing the role of postoperative pain management in recovery. Preoperative pain medication use may indicate more severe or chronic pain conditions, potentially leading to worse surgical outcomes. While cauda equina syndrome was associated with poor outcomes in bivariate analysis, it was not significant in the multivariable model, suggesting that other factors may have a greater impact.

### Strengths and Limitations

We report a large sample of patients from all hospitals in the public health care (wellbeing counties) in Finland. Register-based collecting of the data helps increase the statistical power and allows assessing the association of preoperative factors. In addition, the comprehensive nature of data collection mitigates the impact of selection bias as the catchment area includes the whole country. However, selection may arise from nonresponse and loss to follow-up (attrition bias). Previous studies indicated that outcomes after lumbar discectomy may be worse among the nonresponders than among responders.^17,18^ Nevertheless, the independent data collection process helps diminish various biases, including recall bias and the influence of the diagnostic procedure.

Register-based studies have limitations, including missing values, incomplete patient data, and a lack of control over data collection criteria, which may introduce bias and affect the completeness of analyses. We did not have access to radiologic parameters or extensive information on patient comorbidities. Information on specific type and frequency of preoperative pain medication use was not available. Mental health variables were not available in our data set, which may limit our ability to fully account for psychosocial factors influencing surgical outcomes. Prior research suggests that depression and anxiety contribute to worse pain-related disability.

## CONCLUSIONS

Several preoperative factors were associated with poor outcomes after lumbar discectomy for disc herniation, including older age, higher BMI, and cardiologic comorbidities. These findings highlight the importance of thorough preoperative assessment to identify patients at higher risk. Modifiable factors, such as weight and pain management, should be addressed before surgery when possible. Incorporating these risk factors into preoperative counseling can help manage expectations, support shared decision-making, and potentially improve surgical outcomes.

Key Points

- Older age, female sex, higher BMI, cardiologic comorbidities, prolonged symptoms, preoperative painkiller use, and worse baseline quality of life were associated with higher postoperative disability.
- Employed individuals had better outcomes compared with those on a pension, unemployed, or disabled.
- These findings can guide patient counseling and risk assessment before lumbar discectomy.
